# Measuring Neurobehavioral Disabilities Among Severe Brain Injury Survivors: Reports of Survivors and Proxies in the Chronic Phase

**DOI:** 10.3389/fneur.2019.00051

**Published:** 2019-02-05

**Authors:** Pernille Langer Soendergaard, Lars Siert, Ingrid Poulsen, Rodger Ll. Wood, Anne Norup

**Affiliations:** ^1^Department of Neurology, Rigshospitalet, Glostrup, Denmark; ^2^Department of Psychology, University of Southern Denmark, Odense, Denmark; ^3^RUBRIC (Research Unit on Brain Injury Rehabilitation Copenhagen), Department of Neurorehabilitation, TBI Unit, Copenhagen University Hospital, Rigshospitalet, Hvidovre, Denmark; ^4^Health, Section of Nursing Science, Aarhus University, Aarhus, Denmark; ^5^Swansea University, Institute of Life Sciences, College of Medicine, Swansea, United Kingdom

**Keywords:** neurobehavioral disability, traumatic brain injury, acquired brain injury, SASNOS, chronic phase, proxy ratings

## Abstract

**Background:** Neurobehavioral disability (NBD) has a major influence on long-term psychosocial outcome following acquired brain injury, as it affects not only the survivor of the brain injury, but the whole family.

**Objectives:** To investigate (1) the frequency of NBD among survivors of severe brain injury measured by the Danish version of the St Andrew's-Swansea Neurobehavioural Outcome Scale (SASNOS) rated by patients and proxies, (2) factors associated with NBD, and (3) concordance between reports of NBD completed by patients and proxies.

**Methods:** SASNOS was administered at an outpatient unit as a part of a follow-up assessment after discharge from intensive neurorehabilitation. SASNOS consists of five factors describing the following domains: Interpersonal Behavior, Cognition, Aggression, Inhibition and Communication, and both the patient and a proxy were asked to complete the questionnaire. Data collection was conducted over a period of 2 years, and 32 patients and 31 proxies completed the questionnaire. Mean time since injury was 19.4 months (10.0 SD). Most patients were male (68.8%), and most proxies were female (58.1%). Most of the patients had suffered a traumatic brain injury (68.8%).

**Results:** A fourth of this patient group reported themselves below the normal range on the major domains of Interpersonal Behavior and Cognition. Significant associations between proxies' reports and time since injury, cohabitant status, and the patient's score on the Extended Glasgow Outcome Scale were found. Furthermore, significant differences were found between patient and proxy ratings. Proxies rated patients as having fewer problems on the Interpersonal Behavior domain, and more problems in relation to Cognition. Cognition was the only domain, where patients rated themselves higher indicating fewer problems, compared with their proxies. On both the Aggression and Communication domains, proxies rated patients higher indicating fewer problems than the patients themselves.

**Conclusion:** Danish brain injury survivors experienced NBD as measured by SASNOS. Differences were found between patient and proxy ratings in relation to Cognition and Interpersonal Behavior. The NBDs identified can affect the survivor's ability to reintegrate and participate in activities of daily living, emphasizing how a systematic assessment is required.

## Introduction

Survivors of acquired brain injury (ABI) often experience severe long-term consequences across physical, cognitive, social, behavioral, or psychological domains. Physical or cognitive disabilities can be devastating, but it has been argued that change in neurobehavioral functioning is one of the most distressing legacies of ABI (1, 2). Neurobehavioral disability (NBD) is a term used to describe these neuropsychological and neurological disabilities in behavior amongst ABI survivors (1, 3–5). The concept of NBD was developed to understand and treat the debilitating psychosocial consequences of severe brain injury. NBD comprises weaknesses of attention control, reduced self-awareness, executive dysfunction, lack of insight, problems in social judgements, labile mood, reduced ability to control impulses, and changes in personality (4–6). Poor attentional control can, in itself, contribute to cognitive problems such as difficulties in prospective memory, and executive dysfunctions, contributing to poor self-awareness, difficulties with social judgments, and reduced inhibitory control of emotions and behavior (7–9). Long-term social isolation and poor psychosocial outcome can be a result of these severe consequences (10, 11). Neurobehavioral outcome and the alterations in such behaviors are complex, as they are not only caused by damage to the brain, but also from interaction with the (a) environment, (b) premorbid personality traits, and (c) post injury learning (5, 7, 12, 13). Kreutzer et al. has argued that the presence of NBD is directly associated with poor outcome (10), and Testa et al. found that neurobehavioral problems and impaired family functioning were strongly related (14). However, even though NBD has been shown to be strongly associated with poor outcome in patients and their families (1, 2, 10, 11), increasing caregiver burden and imposing constraints on community independence (15), it is not an easy form of disability to measure, largely because the pattern of disability can be influenced by many components that vary over time. However, it is important to understand these components as they not only affect the long-term wellbeing of the patient, but the whole family (1, 2, 5, 16–18).

The impact of cognitive disabilities in real life situations is not always paralleled by cognitive impairments captured by neuropsychological tests. The structure and composition of tests used in clinical testing mean that some important observations of neurobehavioral disabilities are missed (5, 19–21). The need to identify neuropsychological features of acquired brain injury that are likely to have an adverse psychosocial impact has therefore culminated in a recognition to develop a measure that can capture characteristics of NBD. This includes the ways they affect social functioning and how they interact with the environment and personal traits (12, 13, 19). Furthermore, it has been recognized that a measure of NBD needs to include both a patient and proxy rating, as some patients lack awareness of their disabilities (22).

One method of measuring NBD has been developed by Alderman, Wood and Williams, who introduced St Andrew's-Swansea Neurobehavioral Outcome Scale (SASNOS) (5). SASNOS was specifically designed for patients with ABI and is based on the WHO International Classification of Functioning, Disability and Health (ICF) framework, classifying behavioral problems that have robust psychometric properties. Furthermore, SASNOS was created based on a comprehensive literature review of the existing scales, which highlighted the importance of an instrument being able to identify NBD and long-term psychosocial outcome. SASNOS consists of five factors describing the following domains: Interpersonal Behavior, Cognition, Aggression, Inhibition, and Communication. The raw scores of SASNOS are transformed into T-scores, which can be compared to healthy controls. A T-score < 40 has been used as a clinical cut-off indicating a need for rehabilitation in the specific domain (6). One of the advantages of using SASNOS is that both a patient and a proxy version is available. The proxy version can be completed by both close family members, but also by rehabilitation professionals with comprehensive knowledge about the patient's condition. This is important because some patients experience a lack of awareness of disabilities, thereby underreporting the frequency, severity or significance of specific neurobehavioral problems, compared to proxies (8, 9, 23). Furthermore, studies have indicated that concordance between patient vs. proxy ratings varies across functional domains. Specific items related to self-care or physical function seem to reach high levels of agreement, whereas emotional and behavioral changes seem to be perceived differently by proxies and patients (1, 24, 25). These results support the importance of including an informant or a proxy, e.g., a close relative in the reporting of NBD.

When survivors of severe ABI are seen for follow-up visits after intensive neurorehabilitation, subjective and qualitative reports of NBD by family members frequently occur (25). Due to the lack of ability of standard neuropsychological testing to capture NBD, the present study was designed to quantify these reports systematically by using a validated and reliable measure of NBD. The objectives of the study were to investigate:

- The frequency of NBD among severe brain injury survivors measured by the Danish version of SASNOS as rated by patients and proxies.
- If NBD reports were associated with factors related to patient or proxy.
- Concordance between reports of NBD completed by patients and proxies.

Based on the existing international literature, we hypothesized that the majority of severe ABI survivors would report the presence of NBD in more than one domain, as would their proxies. We hypothesized that factors related to the injury would be associated with both proxies and patients reports of NBD. Furthermore, we hypothesized that discrepancies would be found between reports of patients and proxies, more specifically, that proxies would report more problems than patients in relation to emotional and behavioral disabilities.

## Materials and Methods

### Procedure

After discharge from sub-acute intensive neurorehabilitation in hospital, patients and their close family members were invited for a follow-up visit 1 to 3 years post injury at the outpatient clinic, Department of Neurorehabilitation, TBI Unit, Rigshospitalet, Denmark. During the study period, from December 2015 to December 2017, SASNOS was administered as a part of the standard follow-up assessment. The questionnaire was administered to both the patient and a proxy, in most cases a family member, by a neuropsychologist or a nurse working in the outpatient clinic. The participants were instructed to return the questionnaire when completed, and if the questionnaire was not completed immediately, they were asked to complete it at home and return it in a stamped address envelope.

Patients were included if they met the following criteria:

(1) severe traumatic or non-traumatic brain injury followed by intensive neurorehabilitation at Department of Neurorehabilitation, TBI Unit; (2) ≥18 years at time of follow-up; (3) ≥1year since time of injury; (4) intact ability to understand and read Danish; (5) ≥7 on the Rancho Los Amigos Scale (RLA) at follow-up indicating the resolution of post-traumatic amnesia (PTA) or similar state of confusion for patients with non-traumatic injuries.

For the proxies, the following criteria had to be met: (1) close family member to the patient; (2) ≥18 years at time of follow-up; (3) able to understand and read Danish.

Patients and proxies were excluded if they: (1) had an active substance abuse; (2) had severe aphasia; (3) had severe disorders of consciousness or cognitive disabilities that were too severe to complete the questionnaire.

When the study period was completed, 78 patients had been invited for a follow-up assessment at the outpatient clinic. Of these, 2 never showed up. Of the remaining eligible 76 patients, 18 were excluded due to: aphasia (*n* = 5); not able to understand Danish (*n* = 1); severe cognitive disabilities or disorders of consciousness (*n* = 10); did not have any close proxy (*n* = 1); schizophrenia (*n* = 1). Of the remaining 58 patients who fulfilled our inclusion criteria, a few refused to complete the questionnaire (*n* = 2), but most never returned or received the questionnaire (*n* = 21). Consequently, we received 35 patient ratings. Of these ratings one patient questionnaire was returned anonymously, consequently this was excluded from further analyses. Two patients participated in the follow-up twice and completed the questionnaires both times. Only their first response was included in this study. One patient did not permit his proxy to complete SASNOS, and only the response of the patient was registered. Consequently, we ended up with a sample consisting of 63 questionnaires were included for the analyses, including 32 patient ratings and 31 ratings completed by proxies. This is equal to a response rate of 55.2%.

The study was conducted in concordance with the Helsinki Declaration. Patients and proxies were informed orally and in writing about the purpose of the study and that participation was voluntary before providing consent. Furthermore, that data from the study would be presented in anonymous form without any possibility to recognize the individual participants. Data was handled according to the legislation of the Data Protection Agency, and the Database of Highly Specialized Neurorehabilitation Eastern Denmark has been approved by The Danish Health Data Authority (no. 2012-58-0023).

### Measures

Descriptive data, comprising age at injury, sex, type of injury, time since injury relationship to proxy, and cohabitant status, were collected from the clinic's local database (Database of Highly Specialized Neurorehabilitation Eastern Denmark). Furthermore, length of post-traumatic amnesia (PTA) or confusion was used as an indicator of the severity of the brain injury. A score on the Glasgow Outcome Scale Extended (GOSE) indicated level of global outcome at time of follow-up. If any demographic or injury related data were missing from the local database, the information were retrieved from the patient's file, thereby eliminating any missing data. All questionnaires were investigated for missing data. In case of missing data from one SASNOS subdomain, the mean value, based on the other items from that specific subdomain, was inserted.

PTA/length of confusion: PTA is defined as a period of loss of consciousness and an inability to make consistently new memories after a brain injury. When a patient is consistently oriented and able to remember day to day and make new memories, the resolution of the PTA or confusional state is complete. The length of time a patient remains in PTA or confusional state is a method to assess the severity of the brain injury and is associated with outcome. A duration of PTA of >28 days is considered as a severe brain injury (26, 27).

GOSE: GOSE is an 8-level scale assessing the global outcome after brain injury. The scores indicate: 1 (dead), 2 (vegetative state), 3 (lower severe disability and completely dependent on others), 4 (upper severe disability and some dependency on others, but can be alone for 8 h), 5 (low-moderate disability, living independently, and working at a low level of performance/performing sheltered work), 6 (upper-moderate disability and returning to previous work with adjustments), 7 (low-good recovery with minor consequences of physical or mental deficits), 8 (upper-good recovery, i.e., full functional recovery) (28, 29). It was used to indicate outcome at time of follow-up.

SASNOS: The main outcome measure used in the study was SASNOS, which consists of five major domains measuring NBD following an acquired brain injury: Interpersonal Behavior, Cognition, Inhibition, Aggression, and Communication. Each of the major domains consist of a number of subdomains, which are shown in Table 1.

**Table 1:** Major domains, sub-domains and number of item on SASNOS.

**Major domains**	**Subdomains**	**Number of items**
Interpersonal behavior	Social interaction	5
	Relationships	5
	Engagement	5
Cognition	Executive functioning	6
	Attention and memory	6
Inhibition	Sexual inhibition	3
	Social inhibition	3
Aggression	Provocative behavior	5
	Irritability	4
	Overt aggression	3
Communication	Speech and language	2
	Mental state	2
Total number of items		49

All 49 items are scored on a seven-point Likert-scale from “never” to “always.” Ratings are transformed to standard scores. T-scores with a mean of 50 and standard deviation of 10 is used. Higher scores reflect greater perception of ability and fewer symptoms of neurobehavioral disabilities. Transformation to T-scores allows for cross-scale comparisons and comparisons to neurological healthy individuals. If a patient receives a score of 2 SD (30) from mean of 50 it is statistically significant but if a patient receives a score of 1 SD (31) it is also of clinical interest. Consequently, a clinical cut-off of 40 has been suggested (6). SASNOS consists of two versions; one for proxies or professionals who know the patient well, and another completed by the patient. Good internal consistency has been reported previously with Cronbach's alphas from 0.62 to 0.93 (5) and satisfactory test-retest (0.82–0.96), good inter-rater reliability (0.59–0.83) has also been reported (6).

The questionnaire was translated into Danish following the recommendations for the cross-culture adaption of health status measures, a standardized procedure with back-translation (30) with permission from the original authors (5). Before the start of the present study, the Danish version of SASNOS was piloted by asking 4 patients and 4 proxies to complete SASNOS to investigate if there were any problems in understanding the questions after the translation. This resulted in a modification of the wording in four questions to increase understanding, and these modifications were approved by the original authors.

### Statistical Analysis

Demographics are presented using means and standard deviations (SD) as well as frequencies as appropriate. Ratings on SASNOS were transformed into a standard distribution, and T-scores were calculated using the SASNOS scoring program available online (32). Based on the standardized T-scores, number of ratings below T-score of 40 were calculated. Univariate analyses were applied to investigate associations between factors related to the patient, proxy, injury, and NBD. Furthermore, differences between patient's and proxy's scores were investigated using paired samples *t*-tests. All statistical analyses were conducted using SPSS version 22.0.

## Results

A total of 32 patients and 31 proxies completed and returned SASNOS. The majority of the patients were male (68.8%), and the majority of proxies were female (58.1%). Most proxies were spouses (51.6%) and parents (29.0%) living with the patient (61.3%) (Table 2).

**Table 2:** Demographic characteristics of patients and proxies.

**Characteristics**		**Patient group (*n* = 32)**	**Proxies (*n* = 31)**
		***n*** **(%)**	***n*** **(%)**
Gender	Male	22 (68.8)	13 (41.9)
	Female	10 (31.3)	18 (58.1)
Relationship	Spouse		15 (51.6)
	Parent		9 (29.0)
	Sibling		1 (3.2)
	Child		2 (6.5)
	Close friend		2 (6.5)
	Other type of relative		1 (3.2)
Cohabitants	Yes		19 (61.3)
	No		12 (38.7)

Most brain injury survivors had suffered a traumatic brain injury (TBI; 68.8%) and had a mean age of 44.9 (SD 16.8) at time of the follow-up assessment. Most had sustained a severe injury indicated by length of PTA or period of confusion (Table 3).

**Table 3:** Patient characteristics.

**Characteristics related to the injury**		**Patient group (*n* = 32)**
		***n*** **(%)**
Etiology	TBI	22 (68.8)
	NTBI	10 (31.3)
	Anoxia	2 (6.2)
	Stroke	6 (19.2)
	Meningioma	2 (6.2)
		**Mean (SD)**
Age, time of injury		43.56 (16.92)
Age, follow-up		44.97 (16.80)
Length of PTA(TBI)/Confusion(NTBI) (days)		87.44 (88.02)
Time since injury at follow-up (months)		19.42 (10.02)
GOSE, at follow-up		5.72 (1.44)

*TBI, traumatic brain injury; NTBI, non-traumatic brain injury; GOSE, Glasgow Outcome Scale Extended*.

The mean time since injury was 19.4 months (SD 10.0), and at time of follow-up, only a fourth of the patients were rated as having “good recovery” indicated by GOSE score of 7 or 8.

### Neurobehavioral Disability Measured by SASNOS

Scores outside normal range: Raw scores on SASNOS were transformed to T-scores, and number of patients scoring outside the normal range were investigated. Based on the recommendations by the original authors, normal range was defined as more than 1 SD below the mean (5), and consequently a cut-off of T-score < 40 was used (6). Number of patients rated below the cut-off were calculated (Table 4). Eight patients (25%) rated themselves as below cut-off on the Interpersonal Behavior domain, whereas proxies only rated four patients (12.8%) below the cut-off on the same domain. On the Cognition domain, eight (25%) patients rated themselves below cut-off. On the corresponding rating completed by proxies, 10 patients (32.3%) were rated below the T-score cut-off of 40. On the Aggression, Inhibition and Communication domains, only a few patients were rated below the cut-off.

**Table 4:** Number of patients below cut-off (*T*-score below 40) self-rated or rated by proxies.

	**Patient rating (*n* = 32)** ***n* (%)**	**Proxy rating (*n* = 31)** ***n* (%)**
**Total sum rating**	1 (3.1%)	1 (3.2%)
**Interpersonal Behavior**	8 (25.0%)	4 (12.9%)
**Cognition**	8 (25.0%)	10 (32.3%)
**Aggression**	1 (3.1%)	1 (3.2%)
**Inhibition**	0	0
**Communication**	1 (3.1%)	0

*Number of patients rated with a T-score below 40, on self- or proxy-rating*.

**Figure 1:**
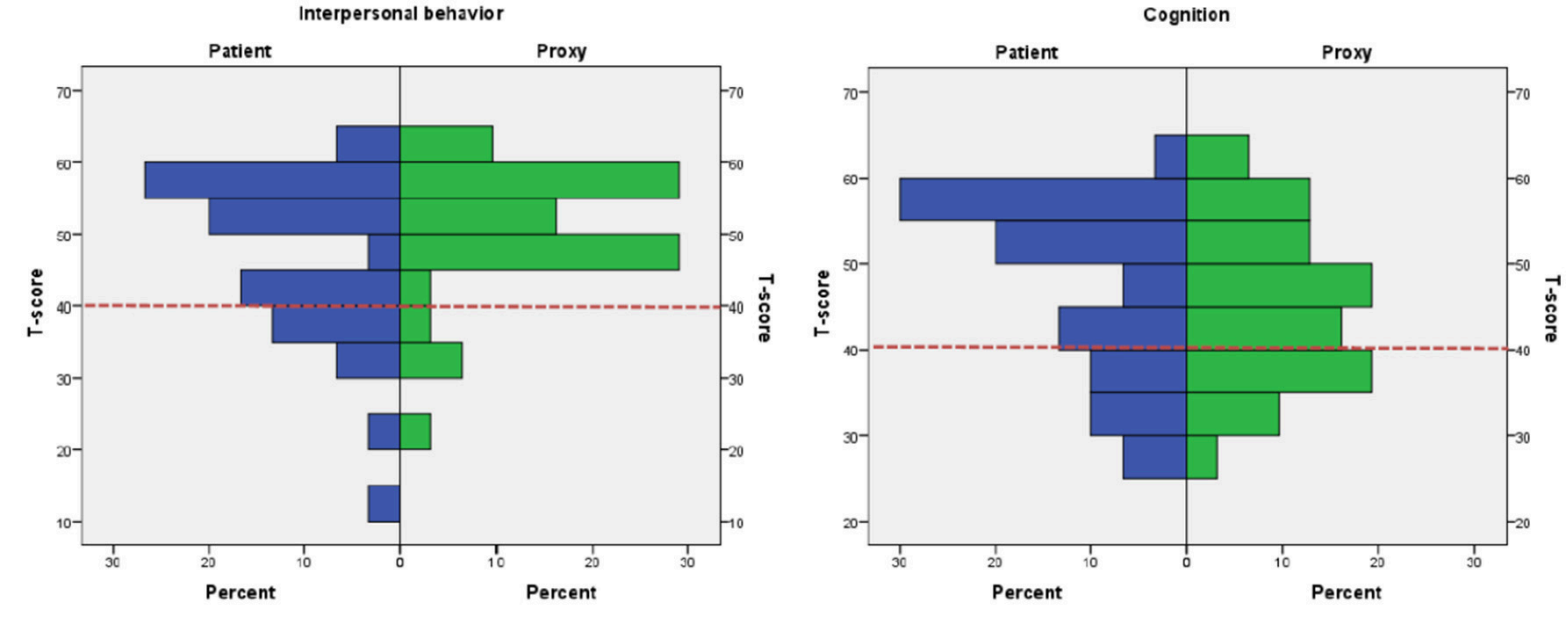
Histograms depicting patient vs. proxy ratings on the major domains Interpersonal Behavior and Cognition. Proxy and patient ratings on Interpersonal Behavior and Cognition. Red dotted line indicates the cut-off of *T*-score 40.

The highest frequencies of patients outside the normal range were found on two major domains of Interpersonal Behavior and Cognition. Ratings are depicted in Figure 1.

### Factors Associated With NBD Ratings

Differences were found in relation to time since injury and cohabitant status. Proxies rated the patients' cognition as significantly lower (*p* = 0.006) the longer the time since injury. Furthermore, proxies living with the patient rated the patient's Interpersonal Behavior (*p* = 0.036) and Aggression (0.044) domains higher, indicating fewer problems, than proxies not living with the patient. Another finding was that there was a significant association between proxies' ratings on the Cognition domain and the patients' scores on GOSE (*p* = 0.001) (Table 5). No differences were found in relation to gender.

**Table 5:** SASNOS domains by proxy and patient characteristics.

**SASNOS**	**All** **Mean (SD)**	**Time since injury** **Mean (SD)**		**Cohabiting** **Mean (SD)**		**GOSE** **Mean (SD)**	
**Main domain**	**Patient (*n* = 31)**	**12 mths**	**>12 mths**	**Yes**	**No**	**3–5**	**6–8**
	**Proxy (*n* = 31)**	**(*n* = 18)**	**(*n* = 13)**	**(*n* = 19)**	**(*n* = 12)**	***n* = 11**	***n* = 20**
**INTERPERSONAL BEHAVIOR**							
Patient	46.42 (11.95)	46.6 (13.3)	47.0 (10.4)	46.5 (11.0)	46.3 (13.7)	45.2 (12.9)	47.7 (11.5)
Proxy	49.71 (9.59)	51.4 (10.4)	48.6 (8.2)	**53.0 (6.2)**	**45.8 (12.2)**^b^	47.5 (8.5)	51.5 (9.9)
**COGNITION**							
Patient	47.56 (10.70)	50.0 (10.7)	45.1 (10.2)	47.6 (11.0)	47.6 (10.8)	46.5 (11.3)	48.6 (10.4)
Proxy	44.36 (8.91)	**49.0 (8.7)**^a^	**40.1 (7.6)**	45.6 (10.1)	44.7 (8.3)	**38.0 (6.7)**	**48.7 (8.4)**^c^
**INHIBITION**							
Patient	61.70 (6.53)	62.8 (6.9)	60.4 (5.7)	63.0 (5.1)	59.7 (8.2)	59.9 (6.6)	62.8 (6.3)
Proxy	61.80 (6.12)	62.7 (6.5)	60.6 (5.6)	62.9 (5.0)	60.0 (7.5)	61.5 (4.3)	62.0 (6.9)
**AGGRESSION**							
Patient	62.34 (9.34)	63.3 (10.2)	61.6 (8.1)	64.6 (5.9)	59.0 (12.5)	59.7 (7.7)	64.2 (9.9)
Proxy	65.40 (7.25)	66.2 (7.5)	64.3 (5.9)	**67.4 (3.6)**	**62.2 (10.0)**^b^	65.5 (6.1)	65.4 (7.7)
**COMMUNICATION**							
Patient	56.32 (8.91)	58.3 (7.6)	53.9 (9.9)	58.4 (7.9)	53.2 (9.7)	54.1 (9.3)	57.7 (8.4)
Proxy	61.80 6.63)	63.0 (5.2)	60.3 (7.9)	62.4 (4)	61.0 (6.4)	63.3 (4.5)	61.2 (7.3)

a p < 0. 01 (p = 0.006);

b p < 0.05 (Interpersonal behavior, p = 0.036); ^b^p < 0.05 (Aggression p = 0.044);

c *p < 0.001 (p = 0.001). Significant differences are marked with bold. GOSE, Glasgow Outcome Scale Extended*.

### Concordance Between Patients' and Proxies' Ratings on Each Domain and Subdomain

**Figure 2:**
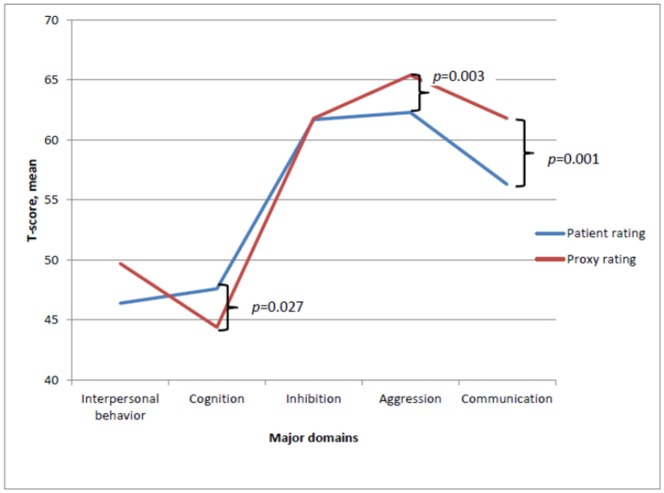
Mean *T*-score ratings completed by patient and proxies on the major domains of SASNOS. Significant difference between patient and proxy ratings on the major domains Cognition, Aggression and Communication.

Differences between ratings completed by proxies and patients were investigated and significant differences were found, both on the Total Sum (*t* = −2.17, df = 30, *p* = 0.040) and on the following domains; Cognition (*t* = 2.33, df = 30, *p* = 0.027), Aggression (*t* = −3.22, df = 30, *p* = 0.003), and Communication (*t* = −3.60, df = 30, *p* = 0.001). The mean scores are depicted in Figure 2. In relation to the domains Aggression and Communication, proxies rated the patients significantly higher, indicating fewer disabilities, whereas the opposite pattern was found on the Cognition domain.

Several subdomains were scored significantly different by patients and proxies, and these are shown in Table 6.

**Table 6:** Patient and proxy ratings on major and subdomains of the SASNOS.

**SASNOS**		**Mean T-score (SD)**	***t*-value**	***p*-value**
**Total sum of ratings**	**Patient**	**54.40 (8.70**	**−2.167**	***0.040***
	**Proxy**	**56.36 (7.29)**		
**Interpersonal behavior**	**Patient**	**46.42 (11.95)**	**−1.798**	**0.083**
	**Proxy**	**49.71 (9.59)**		
Social interaction	Patient	46.72 (8.67)	−0.655	0.517
	Proxy	47.87 (7.81)		
Relationships	Patient	45.65 (14.64)	− 1.974	0.058
	Proxy	49.87 (11.01)		
Engagement	Patient	49.32 (12.60)	− 2.090	*0.046*
	Proxy	52.46 (9.92)		
**Cognition**	**Patient**	**47.56 (10.70)**	**2.331**	***0.027***
	**Proxy**	**44.36 (8.91)**		
Executive functioning	Patient	49.24 (8.95)	2.953	*0.006*
	Proxy	45.02 (9.17)		
Attention and memory	Patient	46.65 (13.42)	0.407	0.687
	Proxy	45.88 (10.75)		
**Inhibition**	**Patient**	**61.70 (6.53)**	**−0.104**	**0.918**
	**Proxy**	**61.80 (6.12)**		
Sexual inhibition	Patient	63.28 (5.02)	− 0.863	0.395
	Proxy	64.00 (4.04)		
Social inhibition	Patient	57.00 (9.40)	0.385	0.703
	Proxy	56.43 (8.43)		
**Aggression**	**Patient**	**62.34 (9.34)**	**−3.217**	***0.003***
	**Proxy**	**65.40 (7.25)**		
Provocative behavior	Patient	62.93 (6.71)	− 1.923	0.064
	Proxy	64.39 (7.08)		
Irritability	Patient	56.22 (12.54)	− 2.927	*0.007*
	Proxy	61.14 (7.82)		
Overt aggression	Patient	63.31 (8.93)	− 2.378	*0.024*
	Proxy	65.62 (5.94)		
**Communication**	**Patient**	**56.32 (8.91)**	**−3.596**	***0.001***
	**Proxy**	**61.80 (6.63)**		
Speech and language	Patient	52.41 (10.86)	− 3.728	*0.001*
	Proxy	59.66 (7.89)		
Mental state	Patient	57.95 (7.89)	− 2.088	*0.046*
	Proxy	60.77 (6.34)		

*Total sum ratings, domains and subdomain are given in the table. Major domains are marked with **bold**. Significant differences between patient and proxy ratings are italized*.

**Figure 3:**
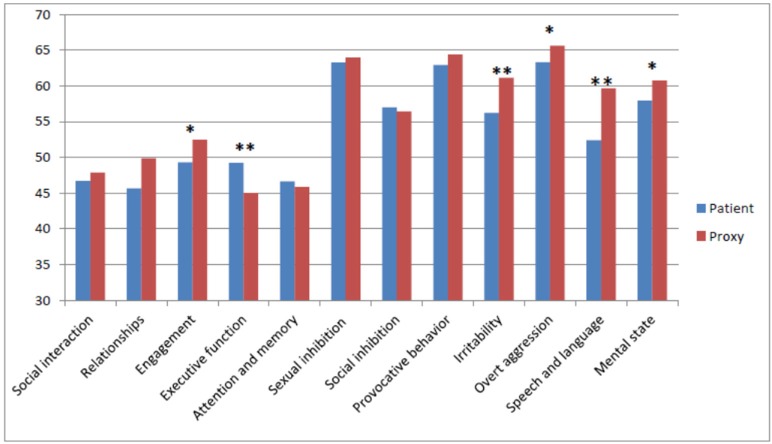
Mean Scores on the subdomains as rated by patients and proxies. Level of significance: ^*^p < 0.05, ^**^p < 0.05.

On a number of subdomains, proxies gave significantly higher ratings; Engagement (*t* = −2.09, df = 30, *p* = 0.046), Irritability (*t* = −2.93, df = 30, *p* = 0.007), Overt aggression (*t* = −2.38, df = 30, *t* = 0.024), Speech and language (*t* = −3.73, df = 30, *p* = 0.001), and Mental state (*t* = −2.09, df = 30, ***p*** **=** 0.046). On the Executive functioning, patients gave significantly higher ratings than proxies (*t* = 2.95, df = 30, *p* = 0.006). These differences are depicted in Figure 3.

As shown in Figure 3, only on the Cognition subdomain, including Executive functioning, and Attention and memory, patients rated themselves higher, indicating fewer problems than considered by the proxies.

## Discussion

This study is the first to report results from the Danish version of SASNOS among proxies and patients with severe ABI. The aim of using SASNOS was to investigate NBD among severe brain injury survivors, factors associated with NBD, and differences between reports of NBD completed by survivors and proxies.

### Frequency of NBD

The results of this study show how a fourth of this patient group reported themselves below the normal range on major domains of Interpersonal Behavior and Cognition. Proxies rated patients as having fewer problems of Interpersonal Behavior and more problems in Cognition. Both major domains have been shown to be sensitive when determining caseness, as they reflect the main problems experienced by ABI survivors. Ratings of symptoms on the remaining three domains Inhibition, Aggression and Communication, were more variable when compared to neurologically healthy adults (5). However, the reported frequencies below cut-off were lower than expected, specifically when considering that the patient group in the current study had suffered a severe ABI, as indicated by the length of confusional state or PTA. Compared to the results reported by Alderman et al. in 2011 and 2017, our patient group scored significantly higher, which indicates fewer problems (5, 6). This was the case for both the patient's self-rating, but also for the proxy ratings. However, a profound difference between our study and the studies conducted by the original authors, is that the patient group in the original studies were rated by professionals working in the rehabilitation setting. It is more than likely that professionals might assess patients differently than a close family member, which served as proxies in our study. Furthermore, the Alderman studies were conducted, respectively 10.5 years (5) and 40.9 months after injury (6). Thus, these studies were completed at a much longer time since injury. Consequently, the patient groups are not completely comparable, which might partly explain the differences. Also, in our group, patients with the most significant NBDs might have been excluded, as they were not able to complete SASNOS independently.

Very recently, the original authors have proposed recalibrating NBD ratings to reflect context-depending support (18). This method would reflect the needed support in relation to each item, consequently in many cases this would assumably lower the obtained ratings.

### Factors Associated With NBD Ratings

We found differences in relation to cohabitant status and NBD ratings. If the proxy and the patient lived together, the proxy tended to rate the patient as having fewer disabilities on the domains of Interpersonal Behavior and Aggression compared to proxies not cohabitating. This finding was in contrast to our expectations, as we expected that proxies in general would report more problems if they lived together, as they would experience the disabilities in activities of daily living first hand. A possible explanation could be a psychological defense mechanism preventing the proxy from acknowledging the disabilities. On the other hand, a proxy not living with the patient, will not have the opportunity to experience the progress in activities of daily living compared to a proxy cohabitating with the patient. One could speculate that this might be why non-cohabitating proxies report more problems. As far as the authors are aware, no studies have specifically investigated cohabitant status and its association with NBD self-reports.

Differences regarding time since injury were also found. Proxies reported significantly more problems on the Cognition domain, the longer time had elapsed since injury. A possible explanation might be the proxy's experience of hopelessness. Early in the rehabilitation process, the proxy may experience optimism in relation to change and spontaneous recovery but, as time goes by, they will have to adapt their life to accommodate the survivor's persisting pattern of disability (33). Contrary to our findings, a SASNOS study, where professionals completed the questionnaire, reported fewer disabilities the longer the time since injury. The largest change in scores was found on the Cognition domain (6). This challenges the belief that neurocognitive functions are static, and how spontaneous recovery might be seen for a longer period than expected (6). However, the design of the mentioned studies was very different with different follow-up periods, and comparative conclusions are difficult to make. Other studies also investigating NBD or neurobehavioral functioning after a brain injury, had fixed time intervals, ranging from discharge (25) to 1 year after injury (1). These fixed follow-up assessments also make it difficult to investigate associations related to time since injury.

Furthermore, we found a significant association between proxy's rating on the Cognition domain and the patient's score on GOSE, indicating more problems when lower GOSE score in obtained compared to a patient with a higher score. A similar association has been reported previously by Holm et al. (25) using the European Brain Injury Questionnaire (EBIQ) and the Glasgow Outcome Scale (GOS) score (25). These findings are not surprising, as lower GOSE scores indicate lower global outcome (28, 29).

### Concordance Between Reports of NBD Completed by Survivors and Proxies

Significant differences were found between patient and proxy ratings. On both the Aggression and Communication domains, proxies rated patients higher, indicating fewer problems than rated by patients themselves. Cognition was the only domain where the patients rated themselves higher, meaning fewer problems compared with their proxies' rating which was in contrast to our expectations. Other studies investigating concordance between patients' and family members' ratings of disabilities have primarily used the Neurobehavioral Functioning Inventory (NFI), and most studies have been conducted in America. Despite differences between the SASNOS and NFI, some of the subscales are similar. NFI also investigates aggression, but where the present study found lower ratings for proxies than patients using SASNOS, Seel et al. found no significant differences on this scale (24). By comparison, Hart el al. found the opposite pattern, namely that proxies rated more problems than patients (1). This might be due to differences in time since injury (1, 24). In the Hart study, only on the Aggression subscale a significant difference was reported between proxy and patient ratings (1). On the Communication scale, the Seel study found a significant difference between the ratings, where patients rated problems on this domain more frequently than proxies did (24). This is in concordance with our study, underlining how patients might perceive this to be a more serious problem than their family members. Another study used EBIQ to investigate complaints following brain injury. They reported no significant differences between patients' and proxies' reports in relation to communication (25).

Cognition was the only domain, where patients reported fewer problems than their proxies. In both the Hart and Seel studies, no significant discrepancies were found between reports on cognition (1, 24). However, the Holm study found a significant difference in relation to cognition, where proxies rated a significantly higher degree of problems than did patients (25). This probably reflects problems with self-awareness, which is often impaired after an acquired brain injury (9). For example, Ciurli et al. noted that poor self-awareness was associated with disabilities in executive functioning (8). Such disability can affect the ability to self-report, as low self-awareness, especially the ability to be aware of one's thoughts and mental state, affects the ability to recognize problems, process, and store information about the self (9). Therefore, lack of insight into one's own disabilities may explain how brain injury survivors sometimes under-report post-injury disabilities (19). As Oddy et al. reported, 40% of family members stated that survivors refused to admit any disabilities following the injury (34). However, discrepancies between survivor and proxy ratings might reflect factors other than decreased self-awareness. For instance, the survivor's communication skills might affect how they are able to communicate such information about disabilities. Furthermore, premorbid personality, relationship to proxy, and the need for compensation or benefits might affect the reports of survivors. As far as the authors are aware, no studies have specifically investigated factors influencing survivors' ability to communicate information about their NBDs. Therefore, it is of great importance to include proxy reports. However, the validity of proxy ratings cannot be guaranteed. Proxies' subjective reports and ratings can be biased and unreliable because of high level of stress associated with trying to cope with changes in their life situation (8), especially when the patient exhibits changes in personality. However, Norup and Mortensen did not find association between personality changes in patients and increased distress in proxies (35). Consequently, whilst patient and proxy reports rely on a subjective evaluation, the method is still of great value to capture cognitive inefficiency in real life situations (5, 19, 20).

It is important to address the long-term impact of NBD and be aware of potential changes in the pattern or degree of disabilities over time. It has become evident that brain injury survivors spend more time at home, have fewer friends and social contacts than prior to the injury (36). Changes in personality (35, 37), cognition and behavior (38, 39) contributes to social handicap. Social isolation can be a consequence of experiencing problems with social interaction (31, 40, 41). Furthermore, due to cognitive disabilities and problems with emotional recognition the survivors might find it difficult to understand why others get upset with them, which can lead to further withdrawal and isolation. This can affect the ability to reintegrate and participate in activities of daily living (42). These consequences emphasize the necessity of a systematic assessment of NBD.

### Study Limitations and Future Perspectives

The present study has some limitations. First of all, it is based on a relatively small sample. Over a period of two years, 32 patients met the inclusion criteria and agreed to participate in the study. The patients included had been hospitalized for specialized neurorehabilitation in the sub-acute phase. A criterion for this type of rehabilitation is that the injury is severe, which was supported by the patients' long period of confusion or PTA. This partly explains why it was not possible to include a larger number of participants. Some of the patients seen for the follow-up assessment in the outpatient clinic had severe cognitive disabilities, disorders of consciousness or severe aphasia. Patients with such disabilities were not able to complete the questionnaire and were consequently excluded. This affects the generalizability of the study, as patients with the most severe injuries might have been excluded. Using data from a cohort at a later stage post injury (> 2 years) could offer a larger sample size. Another reason for the small number of participants could also be a consequence of fatigue, which is common after ABI (43, 44). If a patient felt too exhausted to answer the questionnaire right after the follow-up assessment in the outpatient clinic, they were allowed to complete the questionnaire at home. However, in some cases they might have lacked the motivation to do so or forgot to return the questionnaire. We do not have any information concerning who completed the questionnaire at home or at the outpatient clinic. However, the participants were asked not to discuss their answers prior to completing and returning the questionnaires.

Second, the validity of patient ratings can be challenged if patients with severe brain injury lack the ability to recognize their disabilities. Furthermore, proxy ratings can be biased due to a high level of stress or emotional impact. However, SASNOS has shown good psychometric properties regarding reliability and validity and SASNOS is one way to measure NBD containing the subjective aspect of consequences after ABI.

Third, the single-center design is also a limitation, which warrants caution with respect to generalizing the results. However, as Department of Neurorehabilitation, TBI Unit, covers the Eastern part of Denmark that fact does expand the representativeness of the sample.

The use of SASNOS in this study has indicated areas of potential research. First of all, it could be interesting to explore the impact of the severity of injury in relation to concordance of reports on the SASNOS questionnaires. Second, it could also be of clinical interest to compare SASNOS profiles in cases who have or have not received rehabilitation after ABI. Third, a study exploring if a SASNOS profile at an early stage of recovery can predict psychosocial outcome at a later stage, e.g., 2 to 5 years after injury, would be of clinical interest. If it is possible to identify factors continuously influencing the patient's ability for community reintegration in long-term, it would be possible to focus early or medium-term clinical interventions to help the patient and the family adapt, accommodate, and minimize the social handicap consequent upon NBD. This would be a fruitful area for future research studies, from which both patients and their families would benefit.

## Author Contributions

All authors contributed to conception and design of the study; PS organized the database. AN performed the statistical analysis. PS and AN wrote the first draft of the manuscript. All authors wrote sections of the manuscript and contributed to manuscript revision, read and approved the submitted version.

### Conflict of Interest Statement

The authors declare that the research was conducted in the absence of any commercial or financial relationships that could be construed as a potential conflict of interest.
